# Factors influencing delays in the diagnosis and treatment of bipolar disorder in adolescents and young adults: A systematic scoping review.

**DOI:** 10.1192/j.eurpsy.2024.619

**Published:** 2024-08-27

**Authors:** A. Levit, J.-J. Nunez, E. Morton, K. Keramatian

**Affiliations:** ^1^Department of Psychiatry, University of British Columbia; ^2^Mood Disorders Clinic, Djawad Movafighan Centre for Brain Health, Vancouver, Canada; ^3^School of Psychological Sciences, Monash University, Melbourne, Australia; ^4^Coastal Early Psychosis Intervention Program, North Vancouver, Canada

## Abstract

**Introduction:**

Bipolar Disorder (BD) is a complex psychiatric condition that typically manifests during late adolescence and early adulthood. Over the past two decades, international studies have reported that BD often goes unrecognized and untreated for several years, which can lead to negative clinical and functional outcomes. However, the components of delay in the diagnosis and treatment of BD in adolescents and young adults and various factors influencing those components have not been systematically explored.

**Objectives:**

To determine the known factors that contribute to delays in the treatment of BD in adolescents and young adults and identify current knowledge gaps.

**Methods:**

A conceptual framework based on the *Model of Pathways to Treatment* by Scott and colleagues was used as a foundation for our search and extraction strategy to ensure all components of delay and potential factors influencing each component are explored. We used the Preferred Reporting Items for Systematic Reviews and Meta-Analyses guideline (PRISMA-ScR) to systematically search the electronic databases of MEDLINE (OVID), EMBASE, PsycINFO and CINAHL for peer-reviewed original research articles published from January 01, 2000 through March 29, 2023. Inclusion was restricted to studies with quantitative or qualitative data on individuals diagnosed with bipolar spectrum disorders with symptomatic onset or study participation between the ages of 13-24. Grey literature and studies not published in English were excluded due to resource limitations. Two independent reviewers screened the references retrieved by the literature search based on our inclusion criteria. The findings of included studies were summarized in a narrative and tabular form according to component of delay.

**Results:**

Our search yielded 5180 unique citations, of which 44 articles met our inclusion criteria. We present findings on the patient, illness, and healthcare provider/mental health system factors contributing to the delays in illness appraisal, help-seeking, diagnosis, and treatment.

**Conclusions:**

To the best of our knowledge, this is the first systematic scoping review to explore the potential factors that influence delays in the treatment of BD in adolescents and young adults. Findings from this review will inform clinical practice and policy. We also demonstrate the utility of a systematic approach to identifying the components of delay, from symptom recognition through treatment, as a methodology to help identify knowledge gaps to inform future research.

**Disclosure of Interest:**

None Declared

